# Ion Mobility-Derived Collision Cross-Sections Add Extra Capability in Distinguishing Isomers and Compounds with Similar Retention Times: The Case of Aphidicolanes

**DOI:** 10.3390/md20090541

**Published:** 2022-08-23

**Authors:** Jinmei Xia, Wenhai Xiao, Xihuang Lin, Yiduo Zhou, Peng Qiu, Hongkun Si, Xiaorong Wu, Siwen Niu, Zhuhua Luo, Xianwen Yang

**Affiliations:** 1 Key Laboratory of Marine Biogenetic Resources, Third Institute of Oceanography, Ministry of Natural Resources, Xiamen 361005, China; 2 Key Laboratory of Systems Bioengineering (Ministry of Education), Frontiers Science Center for Synthetic Biology (Ministry of Education), Tianjin University, Tianjin 300072, China; 3 Analyzing and Testing Center, Third Institute of Oceanography, Ministry of Natural Resources, Xiamen 361005, China; 4 Institute of Food Science and Technology, Hebei Agricultural University, Baoding 071001, China

**Keywords:** ion mobility, collision cross-section, natural product, isomer, aphidicolane

## Abstract

The hyphenation of ion mobility spectrometry with high-resolution mass spectrometry has been widely used in the characterization of various metabolites. Nevertheless, such a powerful tool remains largely unexplored in natural products research, possibly mainly due to the lack of available compounds. To evaluate the ability of collision cross-sections (CCSs) in characterizing compounds, especially isomeric natural products, here we measured and compared the traveling-wave IMS-derived nitrogen CCS values for 75 marine-derived aphidicolanes. We established a CCS database for these compounds which contained 227 CCS values of different adducts. When comparing the CCS differences, 36 of 57 pairs (over 60%) of chromatographically neighboring compounds showed a ΔCCS over 2%. What is more, 64 of 104 isomeric pairs (over 60%) of aphidicolanes can be distinguished by their CCS values, and 13 of 18 pairs (over 70%) of chromatographically indistinguishable isomers can be differentiated from the mobility dimension. Our results strongly supported CCS as an important parameter with good orthogonality and complementarity with retention time. CCS is expected to play an important role in distinguishing complex and diverse marine natural products.

## 1. Introduction

Mass spectrometry (MS) is a widely used technique for analyzing various molecules [1]. The development of high-resolution mass spectrometers allows MS to provide more accurate mass-to-charge ratios (*m/z*) of ions. The coupling of ultra-high-performance liquid chromatography (UPLC) with high-resolution time-of-flight (TOF) MS combines high chromatographic resolution with high sensitivity and high mass accuracy. Meanwhile, mass analyzers working in tandem configuration, e.g., quadrupole time-of-flight (QTOF), can offer highly resolved and accurate MS/MS spectra and provide more information for the qualitative analysis of compounds, and greatly improve reliability. The recent advances in mass spectrometry have greatly accelerated the progress of natural product discovery [2].

Hyphenation of MS with ion mobility spectrometry (IMS), IM-MS, can be used in more challenging MS applications, such as in distinguishing isomers [3]. Using IMS, the separation of ions was realized in the gas phase, mainly based on the differences in their charge, shape, and size. The concept of ion mobility can be traced back to the experiments of Rutherford and Thomson in the late 1890s [4], whereas its combination with MS was realized in the 1960s [5]. The electrospray IMS was used as a detection method for HPLC separation in 1998 [6]. A commercially available ion mobility-mass spectrometer, Synapt High-Definition MS, was developed by Waters Corporation in 2006. Drift tube ion mobility spectrometry (DTIMS) and traveling wave ion mobility spectrometry (TWIMS) are two ion mobility techniques that are commonly used. The DTIMS-measured ion drift times can be directly related to collision cross-sections (CCSs) via the Mason–Schamp relationship [7] and this makes DTIMS the only IMS method which can directly measure CCSs. For TWIMS, the construction of the mobility cell is similar to a segmented IMS, and a high field is applied to a segment of the cell and sequentially swept across the cell one section at a time in the direction of ion migration. In this design, the electric field waves pass through the mobility cell and the ions in the cell pass in pulses accordingly and can then be separated based on their different mobilities. The CCS values can be determined via an empirical calibration relationship between measured drift times and known CCS values [8] and this strategy is applicable for both DTIMS and TWIMS. The advances in IMS instrumentation provide unprecedented analytical advantages and enable qualitative and quantitative analysis of various complex samples. The IM-MS is now a powerful technique and has been used in various omics fields [9] (metabolomics [10,11,12,13], lipidomics [14,15,16], proteomics [17], glycomics [18], etc.). IM-MS also plays an important role in the prioritization, discovery, and structure elucidation of secondary metabolites [19].

CCS was believed to be a unique physicochemical parameter that offers a direct reflection of ionic size and configuration in particular gas. In addition to retention time (RT), accurate mass, and MSMS spectrum, the introduction of drift time, or CCS, can greatly improve the reliability of compound identification. CCS can be used as an additional coordinate to further improve confidence in the identification of various chemicals.

The number of CCS values obtained from experiments is limited, whereas various predictive CCS models make obtaining countless theoretical CCS values a reality. Machine learning using artificial neural networks (ANNs) was applied to predict both RT and CCS values [20]. A workflow called the in silico chemical library engine (ISiCLE) has been developed to generate libraries of chemical properties [21]. By using chemical identifiers as input, the probable three-dimensional conformational isomers could be predicted and the CCSs are derived. Based on the algorithm, an online CCS library with over 1 million entries was established (https://metabolomics.pnnl.gov, accessed on 4 January 2022). Using a prediction model called DeepCCS, one can also predict the CCS values via a deep learning algorithm [22]. Ross et al. use molecular quantum numbers (MQNs) to represent the structural characteristics of various molecules [23]. By assessing a variety of machine learning approaches, they established a CCS prediction model (https://CCSbase.net, accessed on 13 April 2022). The software called HPCCS (https://github.com/cepid-cces/hpccs, accessed on 10 April 2022) can be used to calculate the CCSs of both small organic molecules and large protein complexes [24]. Zhu’s group has done a series of work on CCS prediction. They used a support vector regression-based prediction method to predict nitrogen CCS values of metabolites and obtained a predicted CCS database MetCCS [25]. They also developed an approach, LipidCCS, to predict lipid CCS values [26]. They recently reported an ion mobility CCS atlas, namely AllCCS [27], which can be used to predict theoretical CCS values for various small molecules.

Theoretical CCS values can be obtained with high throughput without relying on expensive instruments, while experimental CCS values are always indispensable in verifying the reliability of various theoretical models. Many efforts have been made to obtain experimental CCS values and to form searchable databases. A database for mycotoxins was established which contains more than 100 TWIMS-derived CCS values [28]. The TWIMS-derived CCS values for more than 200 pesticides also formed a database [29]. To aid the identification of metabolites in metabolomics research, the CCSs of 125 common metabolites were measured using TWIMS [30]. An important prerequisite for the applicability of these databases is the reproducibility of the CCS values, especially on different types of instruments and from different laboratories. In the study for the CCS values of pesticides, the CCSs showed high intra- and inter-day repeatability [29]. It is reported that the CCSs were not affected by the complexity of the investigated matrices [28,29]. When various biological matrices (urine, plasma, platelets, red blood cells, etc.) were tested, the CCS measurements showed much better reproducibility than RTs [30]. The CCS values were also shown to be highly reproducible across different instrumental conditions [28] and between instruments located in independent laboratories [30].

The availability of more and more experimental as well as the huge number of predicted CCS data has greatly improved the reliability of compound identification. However, most of the experimental CCS data are from common metabolites [30] or specific types of compounds, such as mycotoxins [28,31], pesticides [29,32], lipids [33], and so on. The experimental CCS information of natural products used to be relatively rare, partly due to the poor availability of natural product molecules, which are always obtained after tedious separation and purification processes. In this regard, the study on drugs and drug-like compounds by Hines et al. greatly enriched the available CCS data for natural products [34].

One of the most important preconceived notions of CCS is that it possesses great potential in discriminating isomers. However, although CCS has been reported to be able to discriminate isomers of certain types of compounds, such as lipids [3], steroids [35], and certain herbal components [36], there has not been an isomer-focused study to evaluate this potential.

**Figure 1:**
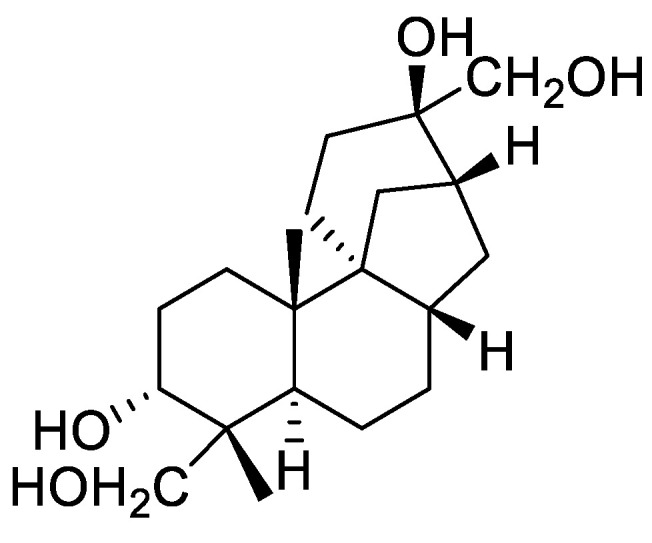
Chemical structure of aphidicolin.

Coincidentally, there are many isomers in natural compounds. For example, in our previous work focusing on the secondary metabolites of a marine-derived fungus [37,38], seven different aphidicolanes corresponding to the same molecular formula of C_22_H_34_O_6_ were obtained, not to mention eight different C_20_H_30_O_5_ molecules and more. Aphidicolin (Figure 1) is a potent DNA polymerase α inhibitor [39] and was explored in clinical trials by the European Organization for Research and Treatment of Cancer [40]. The unique configuration of its functional groups, together with its significant biological activity, makes the discovery of its analogs and the study of their chemistry rather interesting [41]. In this study, as a proof of concept, we explored the potential of CCSs in characterizing marine-derived natural products, especially isomers, using a group of aphidicolane metabolites obtained in our previous work as an example. The CCS values of their different adduct ion forms were obtained using TWIMS, a database was thus established, and the capability of CCSs in distinguishing isomers was evaluated.

## 2. Results and Discussion

To attain the ion mobility information of certain groups of natural compounds, especially isomeric molecules in MS-based analysis, searchable CCS databases are needed. In our previous work, many aphidicolanes were obtained from the solid fermentation of the deep-sea-derived strain *Botryotinia fuckeliana* [37,38]. These compounds were used in this work to establish a CCS database. It was reported that CCSs obtained using direct-infusion MS were consistent with those derived using LC-TWIM-MS analysis [28]. To integrate information from all of the three dimensions, i.e., RT, *m/z*, and CCS values, the UPLC-MS strategy was applied instead of the infusion method in this study. In addition, different forms of adducts were considered when obtaining the CCS values.

### 2.1. Ion Mobility-Derived CCS Values for Aphidicolanes

A lot of efforts have been made to establish and optimize CCS prediction models. Not as much has been done to collect actual CCS values and establish CCS databases. Some CCS libraries are available, which mainly focused on standards and specific types of compounds, such as pesticides [29], metabolites [30], mycotoxins [28], lipids [33], etc. However, the experimentally derived CCS values for natural products are very rare.

In this study, a total of 75 aphidicolanes (Chart S1) were analyzed using LC-TWIM-MS under both positive and negative ionization modes and their CCS values were collected in nitrogen. Among them, reliable signals were obtained for 58 and 61 compounds under positive and negative ionization modes, respectively, of which 44 molecules were detected under both ionization modes. It is also worth mentioning that a large number of dimer ions were also detected for these compounds. A total of 55 of the 58 aphidicolanes for which the [M + Na]^+^ ions were detected also had corresponding [2M + Na]^+^ signals, and a total of 53 of the 61 aphidicolanes which showed [M − H]^−^ adducts were also detected as [2M − H]^−^ ions. The molecular formula, exact mass, RT, type of adduct, and CCS values for all the studied aphidicolanes under ESI+ and ESI− modes are displayed in Tables S3 and S4, respectively (Supporting Information). As shown, the CCS values ranged from 179.88 to 216.29 Å^2^ for the sodium adducts and from 177.27 to 203.31Å^2^ for the deprotonated molecules.

**Figure 2:**
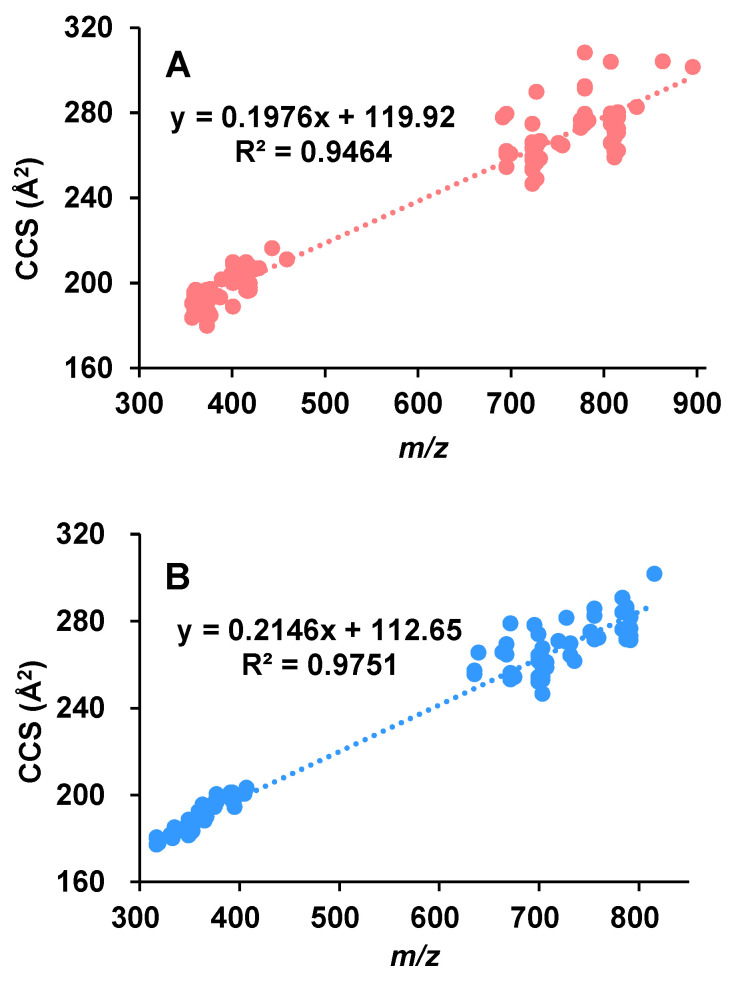
Correlation between the TWIMS-derived CCS values and *m/z* for a series of aphidicolanes. The dimer forms were also included for both the sodium adducts (**A**) and the deprotonated molecules (**B**).

To analyze the CCS-*m/z* correlations, the experimentally determined CCSs were plotted as a function of *m/z* and the corresponding coefficient of determination (*R*^2^) was calculated. Under the ESI+ mode (Figure 2A), both the sodium adducts and their dimer forms were included. Generally speaking, the CCS values tend to increase with the increase of *m/z*. As shown in Figure 2A, the CCS values were strongly correlated (*R*^2^ = 0.9464) with their *m/z* values. It is worth noticing that when the dimers were not considered (Figure S1A), a much smaller *R*^2^ value (0.5513) was obtained, indicating a weaker correlation between the CCSs and their *m/z* values. This is reasonable because many of the aphidicolanes with the same *m/z* in this study have different structures. As shown in Figure S1A, there are several cases where the same *m/z* corresponds to multiple CCSs with large differences. Under the ESI− mode, when the deprotonated molecules and their dimer forms were plotted together with their *m/z* values (Figure 2B), a bigger *R*^2^ value (0.9751) was also obtained, compared with the case where only the deprotonated molecules were considered (Figure S1B, *R*^2^ = 0.8921). The inclusion of the CCS values of dimers led to the increase in *R*^2^ values under both ionization modes. This is partly due to the expansion of the *m/z* range. Another non-negligible reason is that the differences between the CCSs of certain molecules and the differences between their respective dimer CCSs are not strictly proportional. How dimer formation affects the conformations of certain molecules and thus their CCS values needs further study.

**Figure 3:**
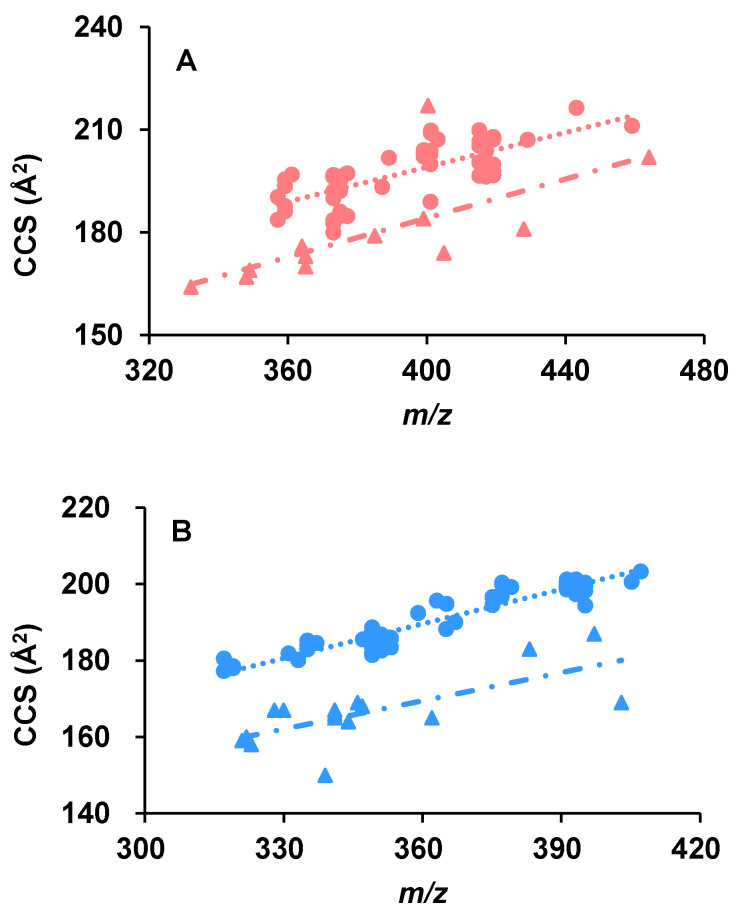
The CCS values of aphidicolanes (filled circles) fall into a different “zone” in the CCS-*m/z* space when compared with the polar metabolites (filled triangles) having similar molecular weight under both positive (**A**) and negative (**B**) ionization modes. The CCS values of the polar metabolites were reproduced with permission from Paglia [30], Ion mobility derived collision cross sections to support metabolomics applications, Analytical Chemistry, 2014.

When the CCS values of the aphidicolanes were plotted together with those of the polar metabolites (data reproduced from the work of Paglia et al. [30] with kind permission), they were noticed to fall in different “zones” (Figure 3). It was reported that the different molecular packing efficiencies in the gas phase can lead to class-specific correlations [42]. According to the work of Paglia et al., the experimentally derived CCS values of metabolites belonging to different chemical classes formed different trend lines. The molecular weight of our aphidicolanes ranges from 334.21 to 436.28 (detected as sodium adducts, *m/z* 357.20 to 459.27) and from 318.18 to 408.25 (detected as deprotonated molecules, *m/z* 317.18 to 407.24) under positive and negative ionization modes, respectively. The CCS values of all the metabolites with a molecular weight from 331.07 to 441.14 (detected as sodium adducts or protonated molecules) and from 322.06 to 404.00 (detected as deprotonated molecules) were used. As different classes of compounds occupy different zones in the CCS-*m/z* space, we can screen certain types of natural products based on their *m/z* and CCS values.

### 2.2. Comparison of Experimental with Theoretical CCS

**Figure 4:**
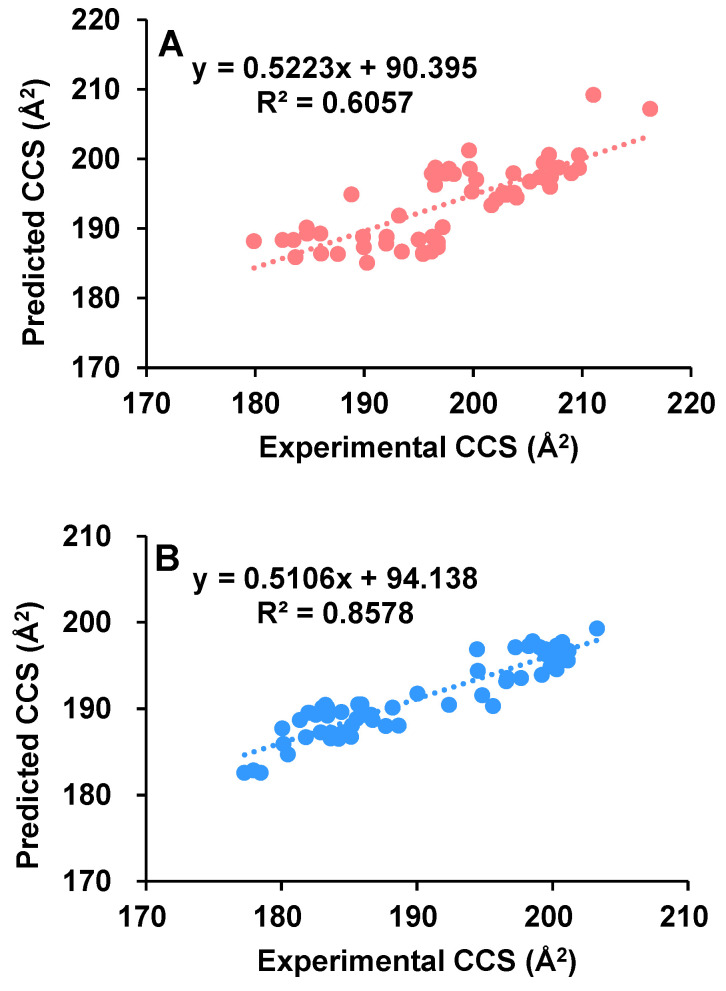
Predicted CCSs using ALLCCS for sodium adducts (**A**) and deprotonated molecules (**B**) of aphidicolanes as a function of experimental CCSs.

To evaluate the performance of in silico models in predicting the CCSs of aphidicolanes, we calculated their theoretical CCSs using ALLCCS [27] as well as CCSbase [23] and compared them with the corresponding experimental CCSs. The AllCCS atlas covers vast chemical structures, and it was demonstrated to be highly accurate in predicting the CCS values for a broad spectrum of small molecules. As in Figure 4, the predicted CCSs were plotted as a function of the experimentally determined CCS values. The deprotonated molecules showed a better correlation between the predicted and experimental CCS values (*R*^2^ = 0.8578) than sodium adducts (*R*^2^ = 0.6057). Reasonable speculation is that the introduction of a sodium ion has a greater effect on the conformation of the molecule and therefore the CCS than losing a proton. Since the predictive model does not take into account the spatial conformation information, its prediction showed larger deviations when dealing with sodium adducts. When CCSbase was used as the predictive model, the linearity was not very good either (Figure S2). This indicates that the deviations between the theoretical and measured CCS are non-negligible. To build a reliable prediction model, it is usually necessary to use available experimental CCS values to train the model. As shown in Figure 3 above, different classes of compounds showed big differences in their CCS values. The lack of enough experimental CCS values of natural products might be the reason for the reduced accuracy of the prediction model when performing such CCS predictions. In addition, for most of the CCS prediction models, the three-dimensional conformation of compounds was not considered, which may also lead to inaccuracy of CCS prediction. The large deviation (the largest was −5.29% in this study) between the predicted and the measured CCS in some cases highlights the necessity of acquiring as many experimental CCS values as possible. In addition, a large number of CCS values of dimers were obtained in this work, which is beyond the capability of current various prediction models.

### 2.3. The CCS Values as an Important Supplementation to the RT Separation

**Figure 5:**
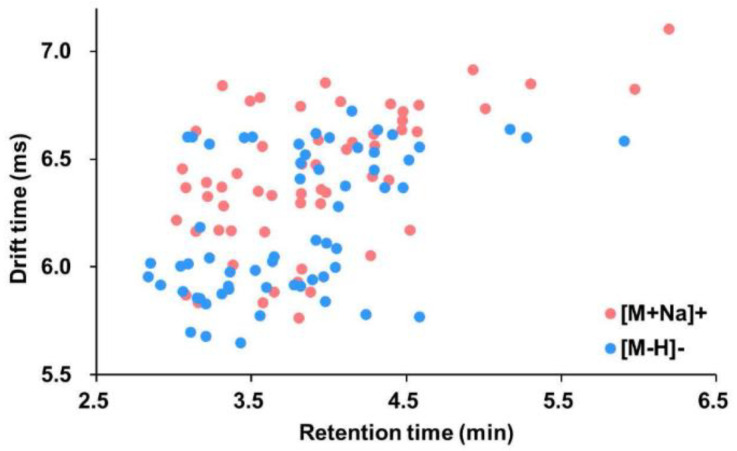
Distribution of sodium adducts and deprotonated molecules of aphidicolanes based on their retention time and drift time.

IM–MS adds a degree of separation to the chromatographic and mass spectrometric detections [43]. To evaluate and compare the distinguishing capabilities of the two types of separation, namely the chromatograph, and the ion mobility, a two-dimensional coordinate system consisting of retention time and drift time was established. Both sodium adducts and deprotonated molecules were then distributed in this space (Figure 5). From the figure, many ions with similar or identical retention times were separated from the drift time dimension. Similarly, a lot of ions with similar or the same drift time were discriminated by their retention time. This illustrates the orthogonal separation effect of drift time and retention time complementing each other.

**Figure 6:**
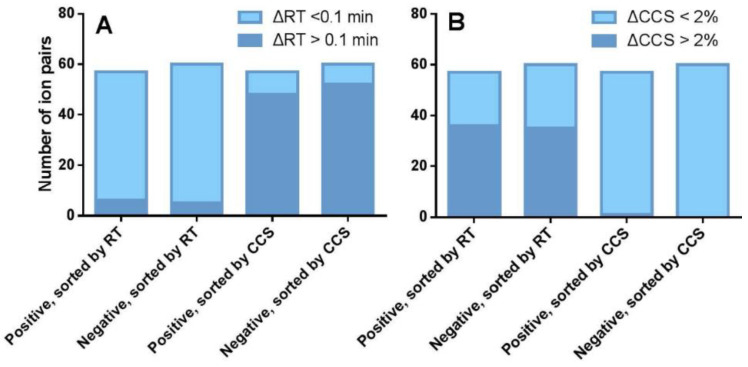
Distribution of aphidicolane molecular pairs according to their RT and CCS differences. (**A**) The number of neighboring ion pairs with RT differences greater and less than 0.1 min when they are sorted according to RT and CCS under both positive and negative ionization modes. (**B**) The number of neighboring ion pairs with CCS differences greater and less than 2% when they are sorted according to RT and CCS under both positive and negative ionization modes.

To evaluate the ability of RT in discriminating aphidicolane ions, all compounds were sorted by RT under positive and negative ionization modes, respectively. The differences in RT between adjacent compounds were then calculated. It was found that the RT differences between most neighbors are less than 0.1 min, which is the typical threshold for RT-based screening. The values ranged from 0.01 to 40.37 s for the sodium adducts whereas for deprotonated molecules from 0.01 to 37.76 s. Among the 57 pairs of chromatographically neighboring compounds under the positive ionization mode, only six pairs showed RT differences greater than 0.1 min (Figure 6A, the first bar on the left). Under the negative ionization mode, 5 out of 60 adjacent compound pairs fulfill the same criteria (Figure 6A, the second bar on the left). Interestingly, when comparing their CCS differences, 36 of 57 (over 60%) and 35 of 60 (nearly 60%) neighboring pairs in the positive and negative ionization modes, respectively, showed CCS differences greater than 2% (Figure 6B, the first two bars on the left), which is a typical criterion for CCS-based screening. This shows that the discrimination on the CCS value dimension is an important supplement to the RT.

To evaluate the ability of ion mobility in discriminating ions, all compounds were sorted by CCSs. The relative CCS differences between every two adjacent compounds were calculated. Similar to the RT dimension, the CCS values for the adjacent aphidicolanes are very close, and the relative CCS differences between most neighbors are less than 2%. In the 57 pairs of comparison for positive and 60 pairs for the negative ionization mode, only one pair of the neighboring compounds in the positive mode showed CCS differences greater than 2% (Figure 6B, the two bars on the right). In the contrast, when comparing their RT difference, 48 of 57 (over 80%) and 52 of 60 (over 80%) neighboring pairs showed RT differences greater than 0.1 min (Figure 6A, the two bars on the right), indicating the role of RT in distinguishing compounds with similar CCSs should not be underestimated.

When comparing the separation capacity of the chromatographic and ion mobility dimensions based on these data, it seems that the RT is more powerful than CCS. One possible reason is that the compounds in this study are purified from a mixture, and the distinguishability in the chromatographic dimension is often the prerequisite for the purification procedure.

### 2.4. The Capability of CCS Values in Discriminating Isomers

For compounds that cannot be separated chromatographically, mass spectrometry provides an ideal extra dimension of separation. As for isomers, ion mobility spectrometry was believed to be a good complement. However, there is no such report on a sufficient number of compounds to evaluate the ability of mobility in distinguishing isomeric ions. As many of the aphidicolanes in this work are isomers, they were used as a case study.

**Figure 7:**
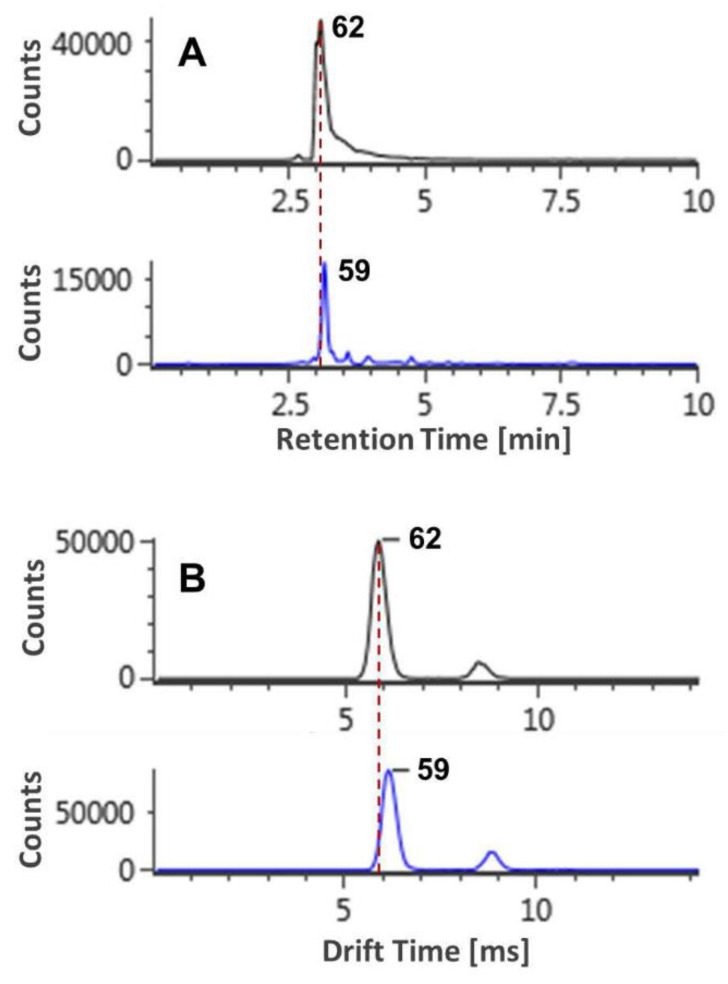

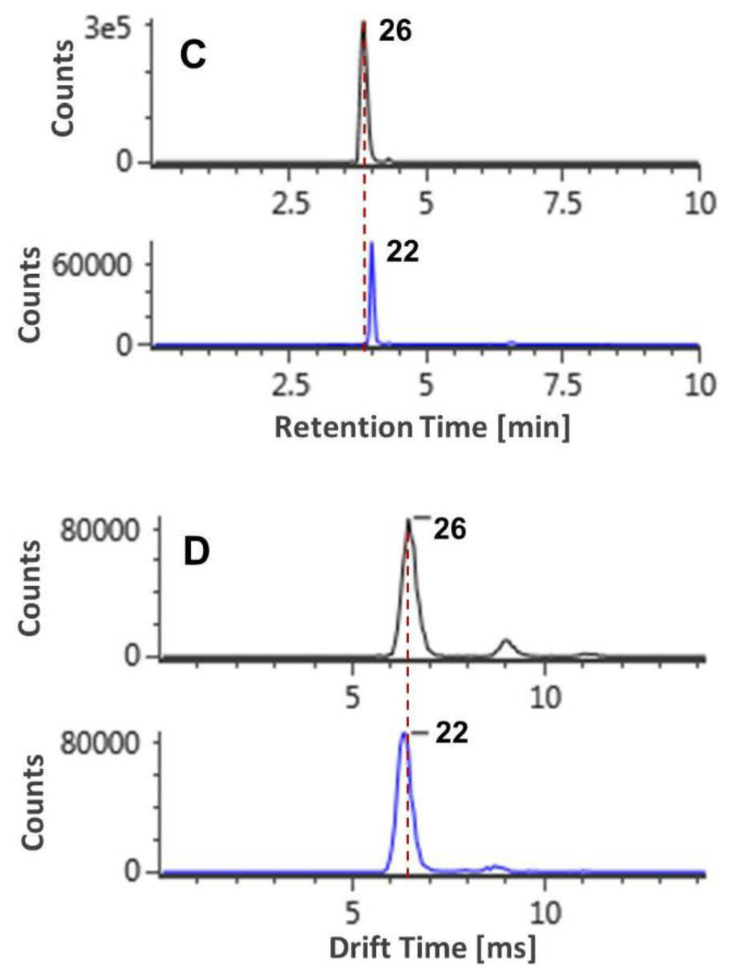
Isomeric compounds **62** and **59** can be discriminated by their CCS values (**B**) but not retention time (**A**), whereas isomers **26** and **22** can be distinguished by retention time (**C**) but showed very similar CCS values (**D**).

When the isomers were closely studied, it was noticed that some were indistinguishable by retention time but could be discriminated from each other by their CCS values. For example, the retention time for compounds **62** and **59** were 3.08 and 3.14 min, respectively (Figure 7A). The difference was less than 0.1 min. In the contrast, their CCS values were 182.52 and 189.98 Å^2^, respectively (Figure 7B). The relative difference was greater than 2%. Quite similarly, also some isomers showed very close CCS values but can be distinguished by their retention time. For example, the CCS values for compounds **26** and **22** were 199.72 and 196.56 Å^2^ and the difference was less than 2% (Figure 7D). Coincidentally, their retention times were 3.82 and 3.95 min, which differentiate them chromatographically (Figure 7C).

Notably, as in Figure 7B,D, there are small peaks alongside the main peaks in the mobility traces of many aphidicolanes. The bimodal distribution was reported for certain antibiotic species, and it was believed to be due to the presence of conformational or isomeric structures or multiple protomers [34]. However, the CCSs of the small peaks here varied a lot from the CCSs of the corresponding main peaks, which made the guess of isomers less convincing. The identities of these small peaks are worth further investigation and beyond the scope of this paper.

To compare the capability of retention times and CCS values in discriminating isomeric pairs of compounds on a larger scale, the isomeric aphidicolanes studied in this work were sorted according to their *m/z* values (Supplementary Tables S5 and S6). For a group of three isomers, when every two compounds were taken as a pair, a total of three isomeric pairs can be formed. As shown in Table S5, the 50 sodium adducts of isomeric aphidicolanes can form 104 isomeric pairs, as there are 12 groups of isomers containing 2, 5, 8, 5, 2, 3, 5, 2, 3, 2, 7, and 6 molecules, respectively. Similarly, the 47 deprotonated molecules listed in Table S6 constitute 89 pairs of isomers.

**Figure 8:**
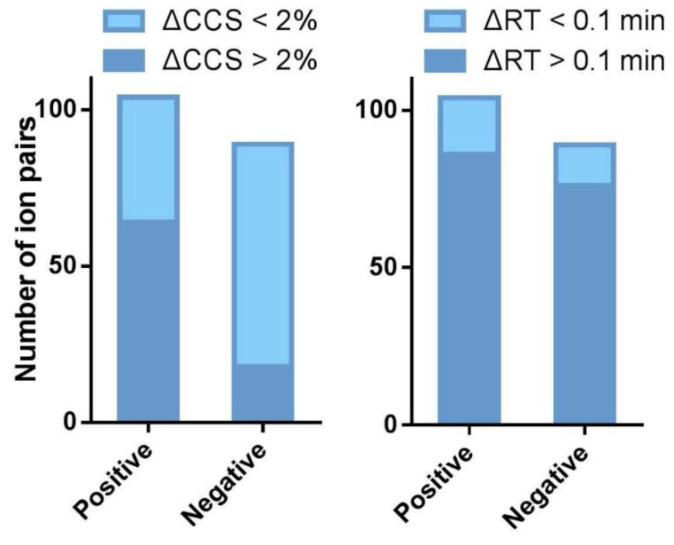
Distribution of isomeric aphidicolane pairs according to their CCS and RT differences under both positive and negative ionization modes.

The differences between RTs and CCS values were then compared between each isomeric pair. The cutoff value for RT and CCS was set as 0.1 min and 2%, respectively. They were taken as the dividing point between good and bad resolutions. In total, 64 of the 104 isomeric aphidicolane pairs in sodium adducts showed ΔCCS values greater than 2%, which accounts for 61.54% (Figure 8). The resolving power of RT was better than CCS values for the sodium ions. Of the 104 isomer pairs, 86 had ΔRT values greater than 0.1 min, which was 82.69% in percentage. The ionization mode showed little influence on the chromatographic separation power. Under the negative ionization mode, 76 of the 89 (85.39%) isomer pairs had RT differences greater than 0.1 min. In the contrast, the differences in CCS values between deprotonated molecules were much smaller than those between sodium adducts. Only 18 out of 89 deprotonated molecules showed CCS differences greater than 2%. The percentage, 20.22%, was much smaller than the 61.545% in the positive mode. It is worth noticing that among the 18 pairs of isomers which cannot be distinguished by their RT, 13 pairs (which account for 72.2%) can be distinguished on CCS (Table S7). These data strongly evidenced that CCS is a parameter with good orthogonality and complementarity with RT.

## 3. Materials and Methods

### 3.1. Chemicals, Reagents, and Materials

All aphidicolanes studied in this work (Chart S1) were obtained from the solid fermentation of the marine fungi *Botryotinia fuckeliana* and were structurally elucidated as described in our previous work [37,38]. They were separately prepared as single reference solutions using 50% methanol in water to reach concentrations of 5~50 µM. Leucine-enkephalin, which was used as the lock mass standard, and the instrument calibration solution (CCS Major Mix) were both purchased from Waters (Manchester, UK). Acetonitrile of LCMS grade was purchased from Merck (Darmstadt, Germany). Ultrapure water (18.2 MΩ cm) was produced using a Milli-Q water purification system.

### 3.2. Liquid Chromatographic Analysis

Waters Acquity UPLC coupled to a TWIMS-QTOFMS instrument (Vion IMS QTOF) was employed. Chromatographic analysis of the aphidicolanes was carried out on an ACQUITY UPLC BEH C18 (50 × 2.1 mm, 1.7 μm) column (part number: 186002350) using an ACQUITY UPLC H-Class system from Waters. Water and acetonitrile were used as mobile phases A and B, respectively. Formic acid was also evaluated during LC-MS analysis but showed no improvement in ionization of the molecular ions; therefore, no buffer was used. The gradient started with a 0.5 min isocratic step at 10% B, ascended to 100% B in 5.5 min and kept for 1 min, and then returned to 10% B in 0.5 min and maintained for 2.5 min. The flow rate was set at 0.3 mL/min. The autosampler temperature was kept at 10 °C and the column temperature at 30 °C; 1 μL of the sample was injected into the system.

### 3.3. MS Analysis

The ESI source was operated in both positive (ESI+) and negative (ESI−) ionization modes. Mass spectra were recorded for *m/z* 50–1000 in the high-definition (HD)MS^E^ mode (which is a data independent acquisition mode) with an acquisition rate of 5 spectra/s. Two independent scans with different collision energies (CE) were alternatively acquired during the run, i.e., a low-energy scan (CE 6 V) to monitor the protonated molecules and other potential adducts and a high-energy scan (CE ramp 25–35 V for ESI+ and 20–30 V for ESI−) to fragment the ions passing through the collision cell. The TOF analyzer was operated in the sensitivity mode. The capillary voltages were set at 3000 V for ESI+ and 2500 V for the ESI− mode, respectively. Nitrogen (>99.5%) was employed as desolvation and cone gas. For the ESI+ mode, the source temperature was 120 °C, and the flow rate of desolvation gas was 800 L/h at 350 °C. In the contrast, 100 °C of source temperature, and 600 L/h of desolvation gas were applied for the ESI− mode.

Calibration of the TOF analyzer was carried out regularly by infusion (10 μL/min) of the corresponding calibration solution according to the manufacturer’s guidelines. For lock mass correction, a 200 ng/mL standard solution of leucine-enkephalin in acetonitrile/water/formic acid (50:49.9:0.1, *v*/*v*/*v*) was continuously infused (5 μL/min) through the reference probe and scanned every 30 s. Data acquisition and processing were performed with UNIFI software (v. 1.8, Waters).

### 3.4. CCS Measurements for Aphidicolanes

CCS calibration was performed regularly using a mixture of calibrants (CCS Major Mix) including singly charged polyalanine oligomers (from *n* = 3 to *n* = 11), leucine-enkephalin, and some other compounds, covering a mass range from 151 to 941 Da (Supporting Information, Table S1) prepared in acetonitrile/water/formic acid (50:49.9:0.1, *v*/*v*/*v*). Argon (≥99.999%) was used as collision-induced dissociation (CID) gas. The parameters used for TWIMS separation were as follows: stopper height, 40 V; trap bias, 40 V; gate height, 40 V; trap wave velocity, 100 m/s; trap pulse height A, 20 V; trap pulse height B, 5 V, IMS wave velocity, 250 m/s; IMS pulse height, 45 V; gate release, 2 ms. Nitrogen (>99.5%) was used as trap and IMS buffer gas at 1.6 L/min and 25 mL/min, respectively.

A randomly picked sample (compound **36**) was used to test the inter-day precisions. The standard deviations of the measured CCS (sodium adducts) and RT values were 0.63 Å^2^ and 0.02 min, respectively (Supporting Information, Table S2).

## 4. Conclusions

In the current study, UPLC coupled to a TWIMS-QTOFMS instrument was applied to obtain more than 220 CCS values of 75 aphidicolanes, which is a group of promising agents with antitumor activities. For 57 pairs of chromatographically neighboring compounds, 51 pairs have RT differences less than 0.1 min and 36 pairs can be distinguished by their CCS values. The aphidicolanes studied showed great structural similarities and formed 104 pairs of isomers. The potential of TWIMS-derived CCS values in distinguishing isomers was thus evaluated. The use of ion mobility in combination with UPLC-MS greatly increased the capacity of distinguishing isomers. A total of 64 of 104 (over 60%) isomeric pairs of aphidicolanes have ΔCCS over 2%. Additionally, 13 of the 18 pairs (over 70%) of chromatographically indistinguishable isomers can be distinguished from each other (ΔCCS > 2%) from the mobility dimension. Using this big group of isomers, the CCS was proved to be an important parameter that has good orthogonality and complementarity with RT. The CCS can be taken as an extra dimension to be included in the natural products screening workflow. This paper provides for the first time a TWIMS-derived CCS database for a large set of marine-derived natural products. The establishment of the database for aphidicolanes that includes CCS, RT, and *m/z* can improve the reliability of screening for aphidicolanes. We encourage further studies to extend the databases of various natural products with CCS values for screening and characterization of secondary metabolites.

## Data Availability

The data presented in this study are available in the Supplementary Materials and can be downloaded at https://www.mdpi.com/article/10.3390/md20090541/s1.

## Supplementary Materials

The following supporting information can be downloaded at: https://www.mdpi.com/article/10.3390/md20090541/s1: Chart S1: Chemical structures of the aphidicolanes studied in this work; Figure S1: Correlation between the TWIMS-derived nitrogen collision cross-sections (CCSs) and *m*/*z* for a series of aphidicolanes when detected as sodium adducts (A) and deprotonated molecules (B); Figure S2: Predicted CCSs using CCSbase for sodium adducts (A) and deprotonated molecules (B) of aphidicolanes as a function of experimental CCSs; Table S1: Collision cross-section (CCS) reference values in nitrogen used for the instrument CCS calibration under positive ([M + H]^+^) and negative ([M − H]^−^) ionization modes; Table S2: The inter-day precision evaluation for compound **36** under positive ionization mode; Table S3: Chromatographic, mass spectrometric, and ion mobility spectrometric information under positive ionization mode for aphidicolanes; Table S4: Chromatographic, mass spectrometric, and ion mobility spectrometric information under negative ionization mode for aphidicolanes; Table S5: Chromatographic, mass spectrometric, and ion mobility spectrometric information for the sodium adducts of aphidicolane isomers; Table S6: Chromatographic, mass spectrometric, and ion mobility spectrometric information for the deprotonated molecules of aphidicolane isomers; Table S7: The relative differences of CCS values for aphidicolane isomers (sodium adducts) which showed RT (retention time) differences less than 0.1 min.
