# Supplementary material for: Ion Mobility-Derived Collision Cross-Sections Add Extra Capability in Distinguishing Isomers and Compounds with Similar Retention Times: The Case of Aphidicolanes
Source: Mar Drugs. 2022 Aug 23;20(9):541. doi: 10.3390/md20090541 (PMC9503386; doi:10.3390/md20090541)
Supplement: Supplementary file 1 [file marinedrugs-20-00541-s001.zip › marinedrugs-1861053-supplementary.pdf]

Chart S1. Chemical structures of the aphidicolin compounds studied in this work.

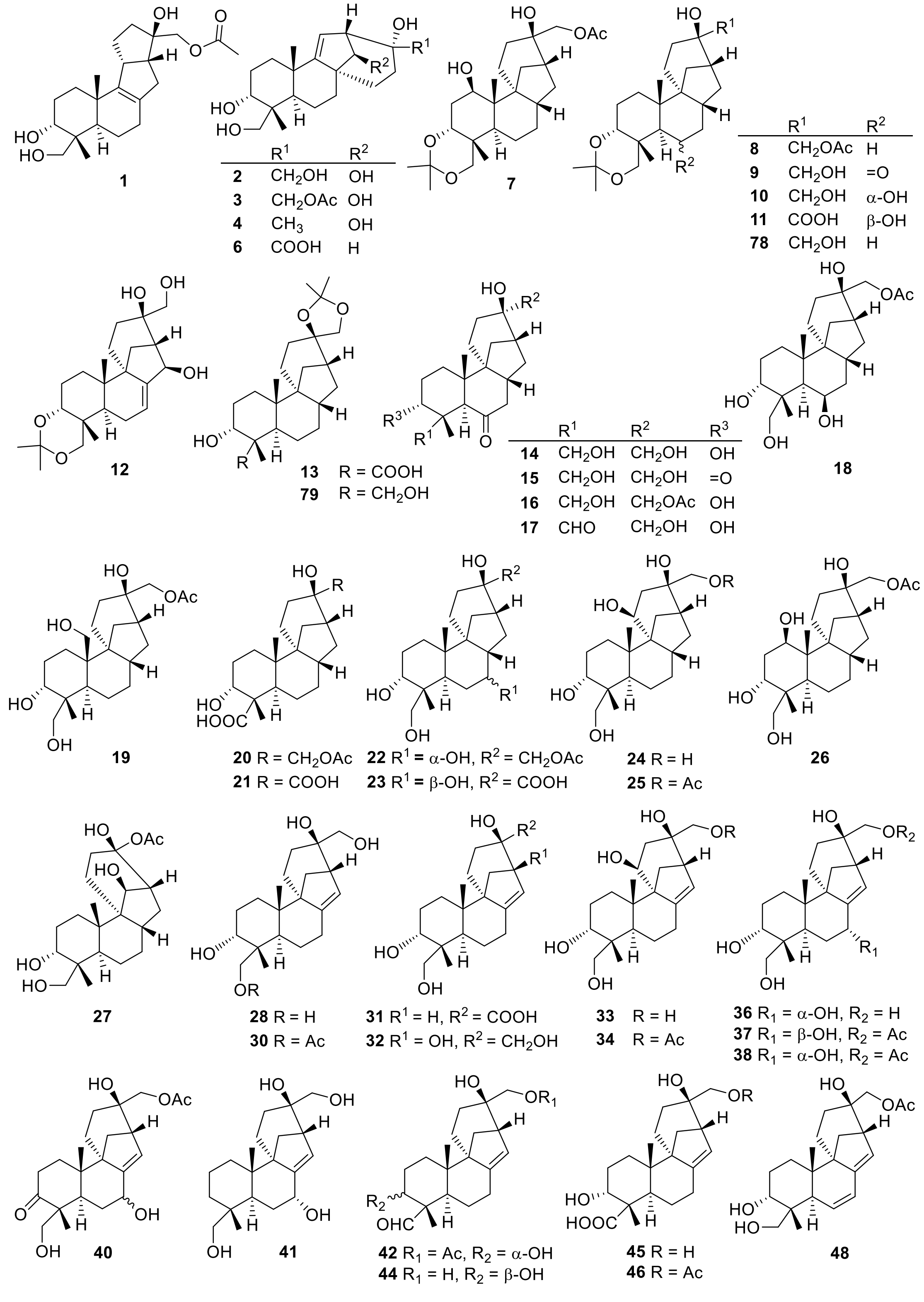

Chart S1. (*cont.*)

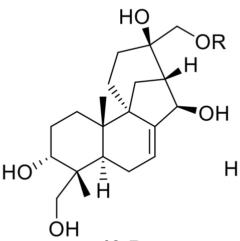

49 R = H
50 R = Ac

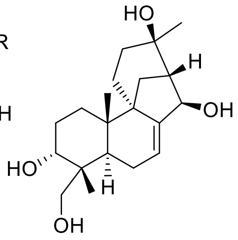

51

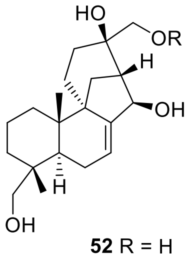

52 R = H
53 R = Ac

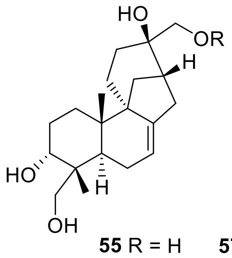

55 R = H
56 R = Ac

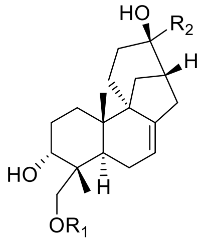

57 R_1 = Ac, R_2 = CH_2OH
58 R_1 = H, R_2 = COOH

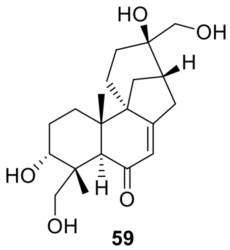

59

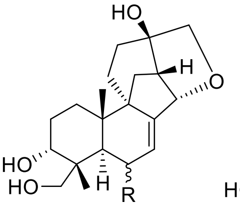

61 R = β-OH
62 R = α-OH

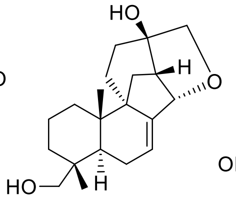

63

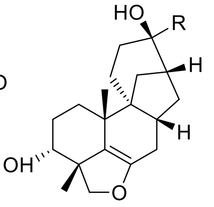

65 R = CH_2OAc
66 R = COOH

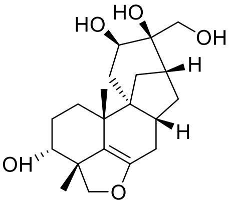

67

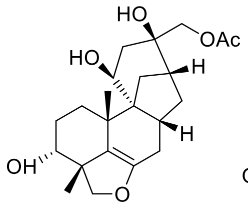

68

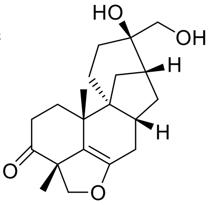

69

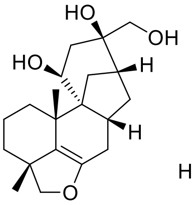

70

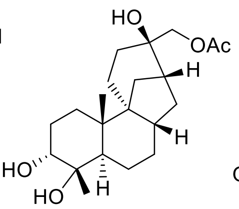

71

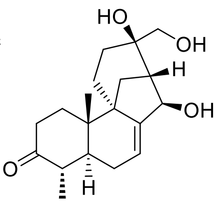

72

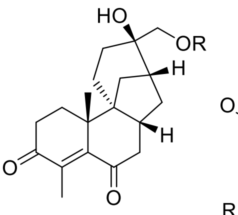

73 R = H
74 R = Ac

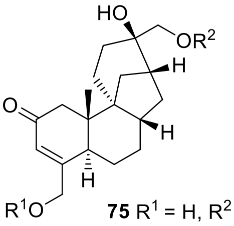

75 R^1 = H, R^2 = H
76 R^1 = Ac, R^2 = H
77 R^1 = Ac, R^2 = Ac

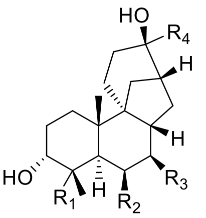

	R^1	R^2	R^3	R^4
**80**	CH_2OH	H	H	CH_2OH
**81**	CH_2OH	H	H	CH_2OAc
**82**	CH_2OH	OH	H	CH_2OH
**83**	COOH	H	H	CH_2OH
**84**	CH_2OH	H	OH	CH_2OH

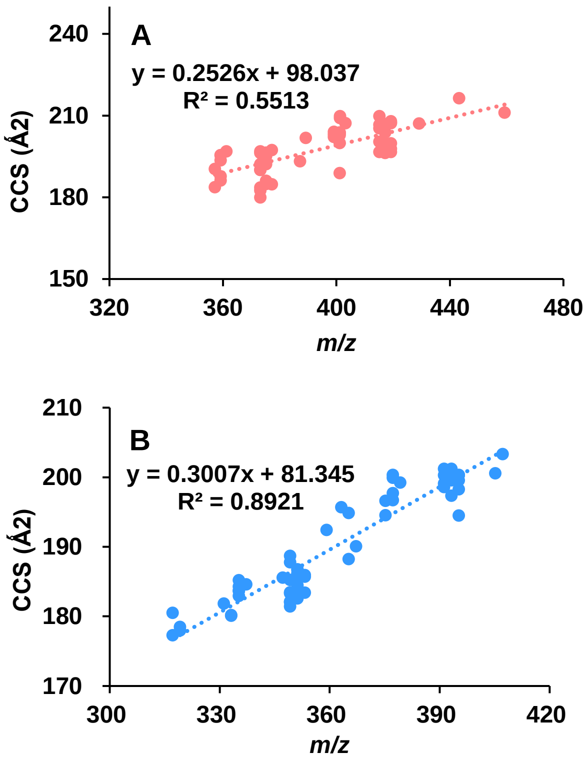

Figure S1. Correlation between the TWIMS-derived nitrogen collision cross-sections (CCSs) and *m/z* for a series of aphidicolin compounds when detected as sodium adducts (A) and deprotonated molecules (B).

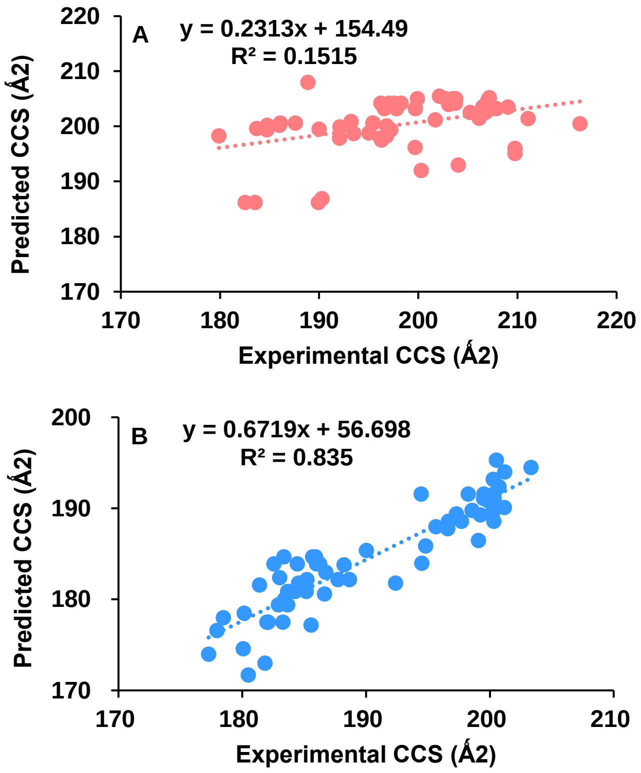

Figure S2. Predicted CCSs using CCSbase[1] for sodium adducts (A) and deprotonated molecules (B) of aphidicolin compounds as a function of experimental CCSs.

Table S1. Collision cross-section (CCS) reference values in nitrogen used for the instrument CCS calibration under positive ($[M+H]^+$) and negative ($[M-H]^-$) ionization modes.

Compound	Formula	Adduct type	Theoretical *m/z* [a]	CCS
Acetaminophen	$C_8H_9NO_2$	$[M+H]^+$	152.07061	130.4
Reserpine fragment	$C_8H_{10}N_4O_2$	$[M+H]^+$	195.08765	138.2
Sulfaguanidine	$C_7H_{10}N_4O_2S$	$[M+H]^+$	215.05972	146.8
Sulfadimethoxine	$C_{12}H_{14}N_4O_4S$	$[M+H]^+$	311.08085	168.4
Val-Tyr-Val	$C_{19}H_{29}N_3O_5$	$[M+H]^+$	380.21800	191.7
Verapamil	$C_{27}H_{38}N_2O_4$	$[M+H]^+$	455.29043	208.8
Terfenadine	$C_{32}H_{41}NO_2$	$[M+H]^+$	472.32101	228.7
Polyalanine, n=7	$C_{21}H_{37}N_7O_8$	$[M+H]^+$	516.27764	211.0
Leucine Enkephalin	$C_{28}H_{37}N_5O_7$	$[M+H]^+$	556.27658	229.8
Polyalanine, n=8	$C_{24}H_{42}N_8O_9$	$[M+H]^+$	587.31475	228.0
Reserpine	$C_{33}H_{40}N_2O_9$	$[M+H]^+$	609.28066	252.3
Polyalanine, n=9	$C_{27}H_{47}N_9O_{10}$	$[M+H]^+$	658.35187	243.0
Polyalanine, n=10	$C_{30}H_{52}N_{10}O_{11}$	$[M+H]^+$	729.38898	256.0
Polyalanine, n=11	$C_{33}H_{57}N_{11}O_{12}$	$[M+H]^+$	800.42609	271.0
Polyalanine, n=12	$C_{36}H_{62}N_{12}O_{13}$	$[M+H]^+$	871.46321	282.0
Polyalanine, n=13	$C_{39}H_{67}N_{13}O_{14}$	$[M+H]^+$	942.50032	294.0
Acetaminophen	$C_8H_9NO_2$	$[M-H]^-$	150.05605	131.5
Theophylline	$C_7H_7N_4O_2$	$[M-H]^-$	179.05745	132.4
Sulfaguanidine	$C_7H_{10}N_4O_2S$	$[M-H]^-$	213.04517	145.2
Sulfadimethoxine	$C_{12}H_{14}N_4O_4S$	$[M-H]^-$	309.06630	170.1
Val-Tyr-Val	$C_{19}H_{29}N_3O_5$	$[M-H]^-$	378.20344	192.5
Leucine Enkephalin	$C_{28}H_{37}N_5O_7$	$[M-H]^-$	554.26202	225.3
Perfluoroheptanoic acid -CO_2	C_6F_{13}	$[M-H]^-$	318.97979	130.1
Perfluorooctanoic acid -CO_2	C_7F_{15}	$[M-H]^-$	368.97660	137.2
Polyalanine, n=8	$C_{24}H_{42}N_8O_9$	$[M-H]^-$	585.30020	227.7
Reserpine	$C_{33}H_{40}N_2O_9$	$[M-H]^-$	607.26610	265.2
Polyalanine, n=9	$C_{27}H_{47}N_9O_{10}$	$[M-H]^-$	656.33731	242.1
Polyalanine, n=10	$C_{30}H_{52}N_{10}O_{11}$	$[M-H]^-$	727.37443	255.9
Polyalanine, n=11	$C_{33}H_{57}N_{11}O_{12}$	$[M-H]^-$	798.41154	268.5
Polyalanine, n=12	$C_{36}H_{62}N_{12}O_{13}$	$[M-H]^-$	869.44865	280.2
Polyalanine, n=13	$C_{39}H_{67}N_{13}O_{14}$	$[M-H]^-$	940.48577	294.6

[a] The theoretical *m/z* are given as neutral species, i.e. the mass of an electron was not accounted for.

Table S2. The inter-day precision evaluation for compound **36** under positive ionization mode.

No.[a]	Molecular formula	Monoisotopic mass	Observed RT [b] (min)	[M+Na]+		[2M+Na]+	
				Theoretical *m/z* [c]	Observed CCS (Å2)	Theoretical *m/z* [c]	Observed CCS (Å2)
**36**	$C_{20}H_{32}O_5$	352.2250	3.38	375.2148	186.00	727.4398	248.82
			3.32		186.24		250.01
			3.37		185.84		249.55
			3.35		185.43		249.60
			3.34		185.61		248.62
			3.35		185.81		248.25
			3.35		185.55		248.69
Standard deviations	-	-	0.02	-	0.28	-	0.64

[a] The numbering of compounds was consistent with Chart S1.

[b] RT, retention time.

[c] The theoretical *m/z* are given as neutral species, i.e. the mass of an electron was not subtracted.

Table S3. Chromatographic, mass spectrometric, and ion mobility spectrometric information under positive ionization mode for aphidicolin compounds.

No.[a]	Molecular formula	Monoisotopic mass	Observed RT (min)	$[M+Na]^+$		$[2M+Na]^+$	
				Theoretical *m/z* [b]	Observed CCS (Å2)	Theoretical *m/z* [b]	Observed CCS (Å2)
**1**	$C_{22}H_{34}O_5$	378.2406	4.27	401.2304	188.86	-	-
**3**	$C_{22}H_{34}O_6$	394.2355	3.14	417.2253	203.73	811.4608	279.02
**7**	$C_{25}H_{40}O_6$	436.2825	4.93	459.2723	211.07	895.5548	301.43
**8**	$C_{25}H_{40}O_5$	420.2876	6.19	443.2774	216.29	863.5650	304.19
**10**	$C_{23}H_{38}O_5$	394.2719	3.92	417.2617	199.66	811.5336	277.49
**12**	$C_{23}H_{36}O_5$	392.2563	3.98	415.2461	209.75	807.5024	303.89
**13**	$C_{23}H_{36}O_5$	392.2563	5.01	415.2461	206.52	807.5024	274.79
**14**	$C_{20}H_{32}O_5$	352.225	3.32	375.2148	195.01	727.4398	264.27
**15**	$C_{20}H_{30}O_5$	350.2093	3.22	373.1991	196.19	723.4084	266.19
**16**	$C_{22}H_{34}O_6$	394.2355	3.82	417.2253	206.80	811.4608	259.06
**17**	$C_{20}H_{30}O_5$	350.2093	3.37	373.1991	192.05	723.4084	274.83
**18**	$C_{22}H_{36}O_6$	396.2512	3.49	419.241	207.35	815.4922	278.59
**19**	$C_{22}H_{36}O_6$	396.2512	3.31	419.241	207.18	815.4922	280.25
**20**	$C_{22}H_{34}O_6$	394.2355	4.48	417.2253	206.14	811.4608	271.57
**22**	$C_{22}H_{36}O_6$	396.2512	3.95	419.241	196.56	815.4922	262.12
**24**	$C_{20}H_{34}O_5$	354.2406	3.88	377.2304	184.74	731.4710	258.38
**25**	$C_{22}H_{36}O_6$	396.2512	4.39	419.241	197.80	815.4922	272.44
**26**	$C_{22}H_{34}O_7$	396.2512	3.82	419.241	199.72	815.4922	270.59
**27**	$C_{22}H_{36}O_6$	396.2512	3.56	419.241	207.86	815.4922	277.78
**28**	$C_{20}H_{32}O_4$	336.2301	3.83	359.2199	187.61	695.4500	262.05
**30**	$C_{21}H_{32}O_5$	378.2406	4.29	401.2304	203.49	779.4710	275.49
**31**	$C_{20}H_{30}O_5$	350.2093	3.81	373.1991	179.88	723.4084	246.54
**32**	$C_{20}H_{32}O_5$	352.225	3.29	375.2148	192.1	727.4398	289.85
**33**	$C_{20}H_{32}O_5$	352.225	3.65	375.2148	184.77	727.4398	258.46
**34**	$C_{22}H_{34}O_6$	394.2355	4.28	417.2253	198.25	811.4608	279.08
**36**	$C_{20}H_{32}O_5$	352.225	3.38	375.2148	186.00	727.4398	248.82
**37**	$C_{22}H_{34}O_6$	394.2355	3.21	417.2253	197.53	811.4608	268.24
**38**	$C_{22}H_{34}O_6$	394.2355	3.82	417.2253	196.22	811.4608	262.26
**40**	$C_{22}H_{32}O_6$	392.2199	3.41	415.2097	196.53	807.4296	265.81
**41**	$C_{20}H_{32}O_4$	336.2301	3.80	359.2199	186.07	695.4500	254.44

Table S3. (*cont.*)

**42**	$C_{22}H_{32}O_5$	376.225	4.48	399.2148	203.07	775.4398	273.48
**44**	$C_{20}H_{30}O_4$	334.2144	3.57	357.2042	183.70	-	-
**45**	$C_{20}H_{30}O_5$	350.2093	3.54	373.1991	196.79	723.4084	257.95
**46**	$C_{22}H_{32}O_6$	392.2199	4.08	415.2097	205.21	807.4296	275.08
**48**	$C_{22}H_{32}O_5$	376.225	4.30	399.2148	202.13	775.4398	276.42
**50**	$C_{22}H_{34}O_6$	394.2355	3.05	417.2253	197.06	-	-
**51**	$C_{20}H_{32}O_4$	336.2301	3.02	359.2199	193.46	695.4500	261.13
**52**	$C_{20}H_{32}O_4$	336.2301	3.31	359.2199	195.42	695.4500	279.51
**53**	$C_{22}H_{34}O_5$	378.2406	3.57	401.2304	199.90	779.4710	291.28
**55**	$C_{20}H_{32}O_4$	336.2301	3.95	359.2199	195.44	695.4500	260.55
**56**	$C_{22}H_{34}O_5$	378.2406	4.57	401.2304	203.78	779.4710	274.81
**57**	$C_{21}H_{32}O_5$	378.2406	3.93	401.2304	202.78	779.4710	308.20
**59**	$C_{20}H_{30}O_5$	350.2093	3.14	373.1991	189.98	723.4084	263.24
**61**	$C_{20}H_{32}O_6$	350.2093	3.16	373.1991	183.54	723.4084	253.98
**62**	$C_{20}H_{30}O_5$	350.2093	3.08	373.1991	182.52	723.4084	253.24
**65**	$C_{22}H_{32}O_5$	376.225	4.47	399.2148	204.02	775.4398	272.91
**67**	$C_{20}H_{30}O_5$	350.2093	3.58	373.1991	189.93	723.4084	259.32
**68**	$C_{22}H_{32}O_6$	392.2199	4.15	415.2097	200.30	807.4296	279.77
**70**	$C_{20}H_{30}O_4$	334.2144	4.52	357.2042	190.28	691.4186	277.79
**71**	$C_{21}H_{34}O_5$	366.2406	4.11	389.2304	201.72	755.4710	264.72
**76**	$C_{21}H_{32}O_5$	364.225	3.82	387.2148	193.21	751.4398	265.81
**77**	$C_{23}H_{34}O_6$	406.2355	4.40	429.2253	207.00	835.4608	282.76
**78**	$C_{23}H_{38}O_4$	378.277	5.30	401.2668	209.74	779.5438	292.37
**79**	$C_{23}H_{38}O_4$	378.277	5.97	401.2668	209.03	779.5438	279.56
**80**	$C_{20}H_{34}O_4$	338.2457	3.98	361.2355	196.79	699.4812	260.65
**81**	$C_{22}H_{36}O_5$	380.2563	4.58	403.2461	207.10	783.5024	276.35
**82**	$C_{20}H_{34}O_5$	354.2406	3.08	377.2304	197.22	731.4710	266.68
**83**	$C_{20}H_{32}O_5$	352.225	3.63	375.2148	196.29	727.4398	256.48

[a] The numbering of compounds was consistent with Chart S1.

[b] The theoretical *m/z* are given as neutral species, i.e. the mass of an electron was not subtracted.

Table S4. Chromatographic, mass spectrometric, and ion mobility spectrometric information under negative ionization mode for aphidicolin compounds.

No.[a]	Molecular formula	Monoisotopic mass	Observed RT (min)	$[M-H]^-$		$[2M-H]^-$	
				Theoretical *m/z* [b]	Observed CCS (Å2)	Theoretical *m/z* [b]	Observed CCS (Å2)
**2**	$C_{20}H_{32}O_5$	352.2250	2.83	351.2172	184.45	703.4422	253.04
**3**	$C_{22}H_{34}O_6$	394.2355	3.12	393.2277	200.28	-	-
**4**	$C_{20}H_{32}O_4$	336.2301	3.06	335.2223	182.92	671.4524	253.14
**6**	$C_{20}H_{30}O_5$	350.2093	3.92	349.2015	188.65	699.4108	273.93
**9**	$C_{23}H_{36}O_5$	392.2563	4.29	391.2485	198.57	783.5048	284.11
**10**	$C_{23}H_{38}O_5$	394.2719	3.92	393.2641	200.72	787.5360	286.52
**11**	$C_{23}H_{36}O_6$	408.2512	4.15	407.2434	203.31	815.4946	301.68
**13**	$C_{23}H_{36}O_5$	392.2563	5.17	391.2485	201.20	783.5048	290.76
**14**	$C_{20}H_{32}O_5$	352.2250	3.35	351.2172	183.02	703.4422	254.70
**16**	$C_{22}H_{34}O_6$	394.2355	3.82	393.2277	197.30	787.4632	271.57
**17**	$C_{20}H_{30}O_5$	350.2093	3.35	349.2015	183.40	699.4108	254.32
**19**	$C_{22}H_{36}O_6$	396.2512	3.45	395.2434	200.26	791.4946	276.54
**20**	$C_{22}H_{34}O_6$	394.2355	4.31	393.2277	201.16	787.4632	285.53
**21**	$C_{20}H_{30}O_6$	366.2042	3.98	365.1964	188.21	731.4006	264.19
**22**	$C_{22}H_{36}O_6$	396.2512	3.85	395.2434	198.26	791.4946	273.06
**23**	$C_{20}H_{32}O_6$	368.2199	3.17	367.2121	190.04	735.4320	261.59
**24**	$C_{20}H_{34}O_5$	354.2406	3.82	353.2328	183.37	707.4734	261.04
**25**	$C_{22}H_{36}O_6$	396.2512	4.36	395.2434	194.45	791.4946	281.75
**26**	$C_{22}H_{34}O_7$	396.2512	3.80	395.2434	199.50	791.4946	271.22
**27**	$C_{22}H_{36}O_6$	396.2512	3.50	395.2434	200.33	791.4946	273.21
**28**	$C_{20}H_{32}O_4$	336.2301	3.78	335.2223	183.66	671.4524	254.82
**32**	$C_{20}H_{32}O_5$	352.2250	3.23	351.2172	186.63	703.4422	261.03
**33**	$C_{20}H_{32}O_5$	352.2250	3.64	351.2172	186.25	703.4422	259.02
**36**	$C_{20}H_{32}O_5$	352.2250	3.31	351.2172	182.54	703.4422	246.63
**37**	$C_{22}H_{34}O_6$	394.2355	3.23	393.2277	199.50	787.4632	275.48
**44**	$C_{20}H_{30}O_4$	334.2144	3.56	333.2066	180.16	667.4210	269.28
**45**	$C_{20}H_{30}O_5$	350.2093	3.52	349.2015	185.21	699.4108	261.72
**46**	$C_{22}H_{32}O_6$	392.2199	4.01	391.2121	200.24	-	-
**48**	$C_{22}H_{32}O_5$	376.2250	4.29	375.2172	196.60	751.4422	275.08
**49**	$C_{20}H_{32}O_5$	352.2250	2.85	351.2172	185.99	703.4422	259.48

Table S4. (*cont.*)

**50**	$C_{22}H_{34}O_6$	394.2355	3.09	393.2277	200.34	787.4632	275.87
**51**	$C_{20}H_{32}O_4$	336.2301	2.91	335.2223	183.68	671.4524	256.21
**52**	$C_{20}H_{32}O_4$	336.2301	3.36	335.2223	185.16	671.4524	278.96
**55**	$C_{20}H_{32}O_4$	336.2301	3.89	335.2223	184.25	671.4524	254.98
**56**	$C_{22}H_{34}O_5$	378.2406	4.52	377.2328	197.70	755.4734	272.22
**57**	$C_{21}H_{32}O_5$	378.2406	3.94	377.2328	196.65	755.4734	282.37
**58**	$C_{20}H_{30}O_5$	350.2093	4.05	349.2015	187.73	699.4108	264.23
**59**	$C_{20}H_{30}O_5$	350.2093	3.20	349.2015	181.38	-	-
**61**	$C_{20}H_{32}O_6$	350.2093	3.15	349.2015	182.08	-	-
**62**	$C_{20}H_{30}O_5$	350.2093	3.17	349.2015	181.98	699.4108	253.00
**63**	$C_{20}H_{32}O_4$	318.2195	4.24	317.2117	180.49	635.4312	255.55
**65**	$C_{22}H_{32}O_5$	376.2250	4.48	375.2172	194.50	-	-
**66**	$C_{20}H_{28}O_5$	348.1937	4.04	347.1859	185.55	695.3796	278.24
**67**	$C_{20}H_{30}O_5$	350.2093	3.60	349.2015	183.28	699.4108	252.01
**68**	$C_{22}H_{32}O_6$	392.2199	4.19	391.2121	199.08	783.4320	276.21
**69**	$C_{20}H_{28}O_4$	332.1988	3.98	331.1910	181.82	663.3898	265.64
**70**	$C_{20}H_{30}O_4$	334.2144	4.59	333.2066	180.07	667.4210	264.63
**71**	$C_{21}H_{34}O_5$	366.2406	4.10	365.2328	194.82	731.4734	269.67
**72**	$C_{19}H_{28}O_4$	320.1988	3.20	319.1910	177.95	639.3898	265.57
**73**	$C_{19}H_{26}O_4$	318.1831	3.43	317.1753	177.27	635.3584	257.22
**74**	$C_{21}H_{28}O_5$	360.1937	4.06	359.1859	192.38	719.3796	270.92
**75**	$C_{19}H_{28}O_4$	320.1988	3.11	319.1910	178.47	-	-
**76**	$C_{21}H_{32}O_5$	364.2250	3.81	363.2172	195.64	727.4422	281.57
**77**	$C_{23}H_{34}O_6$	406.2355	4.41	405.2277	200.54	-	-
**78**	$C_{23}H_{38}O_4$	378.2770	5.28	377.2692	200.31	755.5462	285.68
**79**	$C_{23}H_{38}O_4$	378.2770	5.91	377.2692	199.89	755.5462	271.65
**80**	$C_{20}H_{34}O_4$	338.2457	3.97	337.2379	184.55	675.4836	254.28
**81**	$C_{22}H_{36}O_5$	380.2563	4.59	379.2485	199.22	759.5048	272.31
**82**	$C_{20}H_{34}O_5$	354.2406	3.04	353.2328	185.69	707.4734	258.72
**83**	$C_{20}H_{32}O_5$	352.2250	3.65	351.2172	186.74	703.4422	267.55
**84**	$C_{20}H_{34}O_5$	354.2406	3.09	353.2328	185.92	-	-

[a] The numbering of compounds was consistent with Chart S1.

[b] The theoretical *m/z* are given as neutral species, i.e. the mass of an electron was not accounted for.

Table S5. Chromatographic, mass spectrometric, and ion mobility spectrometric information for the sodium adducts of aphidicolin isomers.

No.[a]	Molecular formula	Monoisotopic mass	Theoretical *m/z* [b]	Observed RT (min)	Observed drift (ms)	Observed CCS (Å2)
**44**	$C_{20}H_{30}O_4$	334.2144	357.2042	3.57	5.83	183.70
**70**	$C_{20}H_{30}O_4$	334.2144	357.2042	4.52	6.17	190.28
**41**	$C_{20}H_{32}O_4$	336.2301	359.2199	3.80	5.93	186.07
**28**	$C_{20}H_{32}O_4$	336.2301	359.2199	3.83	5.99	187.61
**51**	$C_{20}H_{32}O_4$	336.2301	359.2199	3.02	6.22	193.46
**52**	$C_{20}H_{32}O_4$	336.2301	359.2199	3.31	6.37	195.42
**55**	$C_{20}H_{32}O_4$	336.2301	359.2199	3.95	6.29	195.44
**31**	$C_{20}H_{30}O_5$	350.2093	373.1991	3.81	5.76	179.88
**62**	$C_{20}H_{30}O_5$	350.2093	373.1991	3.08	5.87	182.52
**61**	$C_{20}H_{30}O_5$	350.2093	373.1991	3.16	5.83	183.54
**67**	$C_{20}H_{30}O_5$	350.2093	373.1991	3.58	6.16	189.93
**59**	$C_{20}H_{30}O_5$	350.2093	373.1991	3.14	6.16	189.98
**17**	$C_{20}H_{30}O_5$	350.2093	373.1991	3.37	6.17	192.05
**15**	$C_{20}H_{30}O_5$	350.2093	373.1991	3.22	6.33	196.19
**45**	$C_{20}H_{30}O_5$	350.2093	373.1991	3.54	6.35	196.79
**33**	$C_{20}H_{32}O_5$	352.2250	375.2148	3.65	5.88	184.77
**36**	$C_{20}H_{32}O_5$	352.2250	375.2148	3.38	6.01	186.00
**32**	$C_{20}H_{32}O_5$	352.2250	375.2148	3.29	6.17	192.10
**14**	$C_{20}H_{32}O_5$	352.2250	375.2148	3.32	6.28	195.01
**83**	$C_{20}H_{32}O_5$	352.2250	375.2148	3.63	6.33	196.29
**24**	$C_{20}H_{34}O_5$	354.2406	377.2304	3.88	5.88	184.74
**82**	$C_{20}H_{34}O_5$	354.2406	377.2304	3.08	6.37	197.22
**48**	$C_{22}H_{32}O_5$	376.2250	399.2148	4.30	6.56	202.13
**42**	$C_{22}H_{32}O_5$	376.2250	399.2148	4.48	6.68	203.07
**65**	$C_{22}H_{32}O_5$	376.2250	399.2148	4.47	6.63	204.02

Table S5. (*cont.*)

**1**	$C_{22}H_{34}O_5$	378.2406	401.2304	4.27	6.05	188.86
**53**	$C_{22}H_{34}O_5$	378.2406	401.2304	3.57	6.56	199.90
**57**	$C_{22}H_{34}O_5$	378.2406	401.2304	3.93	6.59	202.78
**30**	$C_{22}H_{34}O_5$	378.2406	401.2304	4.29	6.62	203.49
**56**	$C_{22}H_{34}O_5$	378.2406	401.2304	4.57	6.63	203.78
**79**	$C_{23}H_{38}O_4$	378.2770	401.2668	5.97	6.82	209.03
**78**	$C_{23}H_{38}O_4$	378.2770	401.2668	5.30	6.85	209.74
**40**	$C_{22}H_{32}O_6$	392.2199	415.2097	3.41	6.43	196.53
**68**	$C_{22}H_{32}O_6$	392.2199	415.2097	4.15	6.58	200.30
**46**	$C_{22}H_{32}O_6$	392.2199	415.2097	4.08	6.77	205.21
						0.00
**13**	$C_{23}H_{36}O_5$	392.2563	415.2461	5.01	6.73	206.52
**12**	$C_{23}H_{36}O_5$	392.2563	415.2461	3.98	6.85	209.75
**38**	$C_{22}H_{34}O_6$	394.2355	417.2253	3.82	6.34	196.22
**50**	$C_{22}H_{34}O_6$	394.2355	417.2253	3.05	6.45	197.06
**37**	$C_{22}H_{34}O_6$	394.2355	417.2253	3.21	6.39	197.53
**34**	$C_{22}H_{34}O_6$	394.2355	417.2253	4.28	6.42	198.25
**3**	$C_{22}H_{34}O_6$	394.2355	417.2253	3.14	6.63	203.73
**20**	$C_{22}H_{34}O_6$	394.2355	417.2253	4.48	6.72	206.14
**16**	$C_{22}H_{34}O_6$	394.2355	417.2253	3.82	6.74	206.80
**22**	$C_{22}H_{36}O_6$	396.2512	419.2410	3.95	6.36	196.56
**25**	$C_{22}H_{36}O_6$	396.2512	419.2410	4.39	6.40	197.80
**26**	$C_{22}H_{36}O_6$	396.2512	419.2410	3.82	6.48	199.72
**19**	$C_{22}H_{36}O_6$	396.2512	419.2410	3.31	6.84	207.18
**18**	$C_{22}H_{36}O_6$	396.2512	419.2410	3.49	6.77	207.35
**27**	$C_{22}H_{36}O_6$	396.2512	419.2410	3.56	6.78	207.86

[a] The numbering of compounds was consistent with Chart S1.

[b] The theoretical *m/z* are given as neutral species, i.e. the mass of an electron was not subtracted.

Table S6. Chromatographic, mass spectrometric, and ion mobility spectrometric information for the deprotonated molecules of aphidicolin isomers.

No.[a]	Molecular formula	Monoisotopic mass	*m/z* [b]	Observed RT (min)	Observed drift (ms)	Observed CCS (Å2)
**75**	$C_{19}H_{28}O_4$	320.1988	319.1910	3.11	5.70	178.47
**72**	$C_{19}H_{28}O_4$	320.1988	319.1910	3.20	5.68	177.95
**70**	$C_{20}H_{30}O_4$	334.2144	333.2066	4.59	5.77	180.07
**44**	$C_{20}H_{30}O_4$	334.2144	333.2066	3.56	5.77	180.16
**28**	$C_{20}H_{32}O_4$	336.2301	335.2223	3.78	5.92	183.66
**51**	$C_{20}H_{32}O_4$	336.2301	335.2223	2.91	5.92	183.68
**4**	$C_{20}H_{32}O_4$	336.2301	335.2223	3.06	5.89	182.92
**55**	$C_{20}H_{32}O_4$	336.2301	335.2223	3.89	5.94	184.25
**52**	$C_{20}H_{32}O_4$	336.2301	335.2223	3.36	5.98	185.16
**59**	$C_{20}H_{30}O_5$	350.2093	349.2015	3.20	5.83	181.38
**58**	$C_{20}H_{30}O_5$	350.2093	349.2015	4.05	6.09	187.73
**6**	$C_{20}H_{30}O_5$	350.2093	349.2015	3.92	6.12	188.65
**62**	$C_{20}H_{30}O_5$	350.2093	349.2015	3.17	5.85	181.98
**17**	$C_{20}H_{30}O_5$	350.2093	349.2015	3.35	5.91	183.40
**45**	$C_{20}H_{30}O_5$	350.2093	349.2015	3.52	5.98	185.21
**67**	$C_{20}H_{30}O_5$	350.2093	349.2015	3.60	5.91	183.28
**61**	$C_{20}H_{30}O_5$	350.2093	349.2015	3.15	5.86	182.08
**14**	$C_{20}H_{32}O_5$	352.2250	351.2172	3.35	5.90	183.02
**49**	$C_{20}H_{32}O_5$	352.2250	351.2172	2.85	6.02	185.99
**33**	$C_{20}H_{32}O_5$	352.2250	351.2172	3.64	6.03	186.25
**36**	$C_{20}H_{32}O_5$	352.2250	351.2172	3.31	5.88	182.54
**2**	$C_{20}H_{32}O_5$	352.2250	351.2172	2.83	5.95	184.45
**83**	$C_{20}H_{32}O_5$	352.2250	351.2172	3.65	6.05	186.74
**32**	$C_{20}H_{32}O_5$	352.2250	351.2172	3.23	6.04	186.63
**82**	$C_{20}H_{34}O_5$	354.2406	353.2328	3.04	6.00	185.69
**24**	$C_{20}H_{34}O_5$	354.2406	353.2328	3.82	5.91	183.37
**84**	$C_{20}H_{34}O_5$	354.2406	353.2328	3.09	6.01	185.92

Table S6. (*cont.*)

**65**	$C_{22}H_{32}O_5$	376.2250	375.2172	4.48	6.37	194.50
**48**	$C_{22}H_{32}O_5$	376.2250	375.2172	4.29	6.45	196.60
**56**	$C_{22}H_{34}O_5$	378.2406	377.2328	4.52	6.50	197.70
**57**	$C_{22}H_{34}O_5$	378.2406	377.2328	3.94	6.45	196.65
**78**	$C_{23}H_{38}O_4$	378.2770	377.2692	5.28	6.60	200.31
**79**	$C_{23}H_{38}O_4$	378.2770	377.2692	5.91	6.58	199.89
**68**	$C_{22}H_{32}O_6$	392.2199	391.2121	4.19	6.55	199.08
**46**	$C_{22}H_{32}O_6$	392.2199	391.2121	4.01	6.60	200.24
**9**	$C_{23}H_{36}O_5$	392.2563	391.2485	4.29	6.53	198.57
**13**	$C_{23}H_{36}O_5$	392.2563	391.2485	5.17	6.64	201.20
**37**	$C_{22}H_{34}O_6$	394.2355	393.2277	3.23	6.57	199.50
**16**	$C_{22}H_{34}O_6$	394.2355	393.2277	3.82	6.48	197.30
**20**	$C_{22}H_{34}O_6$	394.2355	393.2277	4.31	6.64	201.16
**50**	$C_{22}H_{34}O_6$	394.2355	393.2277	3.09	6.60	200.34
**3**	$C_{22}H_{34}O_6$	394.2355	393.2277	3.12	6.60	200.28
**22**	$C_{22}H_{36}O_6$	396.2512	395.2434	3.85	6.52	198.26
**25**	$C_{22}H_{36}O_6$	396.2512	395.2434	4.36	6.37	194.45
**19**	$C_{22}H_{36}O_6$	396.2512	395.2434	3.45	6.60	200.26
**27**	$C_{22}H_{36}O_6$	396.2512	395.2434	3.50	6.60	200.33
**26**	$C_{22}H_{36}O_6$	396.2512	395.2434	3.80	6.57	199.50

[a] The numbering of compounds was consistent with Chart S1.

[b] The theoretical *m/z* are given as neutral species, i.e. the mass of an electron was not accounted for.

Table S7. The relative differences of CCS values for aphidicolin isomers (sodium adducts) which showed RT (retention time) differences less than 0.1 min.

No.[a]	Molecular formula	Theoretical *m/z* [b]	Observed RT (min)	Observed CCS (Å2)	ΔRT (min)	ΔCCS
**1**	$C_{22}H_{34}O_5$	401.2304	4.27	188.86	0.02	7.75%
**30**	$C_{22}H_{34}O_5$	401.2304	4.29	203.49		
**3**	$C_{22}H_{34}O_6$	417.2253	3.14	203.73	0.07	3.14%
**37**	$C_{22}H_{34}O_6$	417.2253	3.21	197.53		
**3**	$C_{22}H_{34}O_6$	417.2253	3.14	203.73	0.09	3.39%
**50**	$C_{22}H_{34}O_6$	417.2253	3.05	197.06		
**14**	$C_{20}H_{32}O_5$	375.2148	3.32	195.01	0.03	1.52%
**32**	$C_{20}H_{32}O_5$	375.2148	3.29	192.10		
**14**	$C_{20}H_{32}O_5$	375.2148	3.32	195.01	0.06	4.85%
**36**	$C_{20}H_{32}O_5$	375.2148	3.38	186.00		
**15**	$C_{20}H_{30}O_5$	373.1991	3.22	196.19	0.08	3.27%
**59**	$C_{20}H_{30}O_5$	373.1991	3.14	189.98		
**15**	$C_{20}H_{30}O_5$	373.1991	3.22	196.19	0.06	6.89%
**61**	$C_{20}H_{30}O_5$	373.1991	3.16	183.54		
**16**	$C_{22}H_{34}O_6$	417.2253	3.82	206.80	0.00	5.39%
**38**	$C_{22}H_{34}O_6$	417.2253	3.82	196.22		
**18**	$C_{22}H_{36}O_6$	419.2410	3.49	207.35	0.07	0.25%
**27**	$C_{22}H_{36}O_6$	419.2410	3.56	207.86		
**28**	$C_{20}H_{32}O_4$	359.2199	3.83	187.61	0.03	0.83%
**41**	$C_{20}H_{32}O_4$	359.2199	3.80	186.07		
**32**	$C_{20}H_{32}O_5$	375.2148	3.29	192.10	0.09	3.28%
**36**	$C_{20}H_{32}O_5$	375.2148	3.38	186.00		
**33**	$C_{20}H_{32}O_5$	375.2148	3.65	184.77	0.01	6.23%
**83**	$C_{20}H_{32}O_5$	375.2148	3.63	196.29		

Table S7. (*cont.*)

**42**	$C_{22}H_{32}O_5$	399.2148	4.48	203.07	0.00	0.47%
**65**	$C_{22}H_{32}O_5$	399.2148	4.47	204.02		
**45**	$C_{20}H_{30}O_5$	373.1991	3.54	196.79	0.04	3.61%
**67**	$C_{20}H_{30}O_5$	373.1991	3.58	189.93		
**46**	$C_{22}H_{32}O_6$	415.2097	4.08	205.21	0.08	2.46%
**68**	$C_{22}H_{32}O_6$	415.2097	4.15	200.30		
**59**	$C_{20}H_{30}O_5$	373.1991	3.14	189.98	0.07	4.08%
**62**	$C_{20}H_{30}O_5$	373.1991	3.08	182.52		
**59**	$C_{20}H_{30}O_5$	373.1991	3.14	189.98	0.02	3.50%
**61**	$C_{20}H_{30}O_5$	373.1991	3.16	183.54		
**61**	$C_{20}H_{30}O_5$	373.1991	3.16	183.54	0.08	0.56%
**62**	$C_{20}H_{30}O_5$	373.1991	3.08	182.52		

[a] The numbering of compounds was consistent with Chart S1.

[b] The theoretical *m/z* are given as neutral species, i.e. the mass of an electron was not subtracted.

Reference:

1. Ross, D. H.; Cho, J. H.; Xu, L., Breaking down Structural Diversity for Comprehensive Prediction of Ion-Neutral Collision Cross Sections. *Analytical Chemistry* **2020,** *92*, 4548-4557.
